# Comprehensive Geriatric Assessment Domains and Routine Inflammatory Indices in Relation to Clinical Frailty Scale-Defined Frailty: A Cross-Sectional Study in Hospital-Based Older Adults

**DOI:** 10.3390/healthcare14203375

**Published:** 2026-10-10

**Authors:** Latife Merve Yıldız, Handan Duman, Damla Tüfekçi

**Affiliations:** 1 Department of Family Medicine, Faculty of Medicine, Ordu University, 52200 Ordu, Türkiye; 2 Department of Family Medicine, Faculty of Medicine, Recep Tayyip Erdoğan University, 53100 Rize, Türkiye; 3 Division of Endocrinology and Metabolic Diseases, Department of Internal Medicine, Faculty of Medicine, Recep Tayyip Erdoğan University, 53100 Rize, Türkiye; drtufekci@gmail.com

**Keywords:** frailty, geriatric assessment, biomarkers, primary health care, family practice

## Abstract

Background/Objectives: Frailty in older adults is multidimensional, but the added value of routine blood-based inflammatory indices beyond comprehensive geriatric assessment is uncertain. In a hospital-based sample we examined how Clinical Frailty Scale (CFS)-defined frailty relates to geriatric domains and to inflammatory/prognostic indices, and whether these indices improve the cross-sectional classification of frailty beyond a core geriatric model. Methods: In this single-center, cross-sectional study, 284 of 300 consecutive records of adults aged 65 years or older who underwent comprehensive geriatric assessment at a family medicine-run healthy aging outpatient clinic in a university hospital were analyzed after data-quality checks. Frailty was defined as a CFS score of 5 or higher. Function, nutrition, cognition, mood, and mobility were assessed with Katz Activities of Daily Living (ADL), Lawton Instrumental Activities of Daily Living (IADL), Mini Nutritional Assessment (MNA), Mini-Cog, Geriatric Depression Scale-15, and Timed Up and Go (TUG). Five inflammatory/prognostic indices, including the platelet-to-lymphocyte ratio (PLR), were recalculated from same-day laboratory values. Logistic regression and receiver operating characteristic analyses compared a basic clinical model, a core geriatric model, and index-added models, with bootstrap assessment of calibration and optimism. Results: Frailty prevalence was 54.6% (95% confidence interval [CI]: 48.8–60.3). The area under the curve was 0.611 in the basic clinical model and 0.866 (95% CI: 0.821–0.907) in the core geriatric model (optimism-corrected 0.842; calibration slope 0.81). Because several geriatric measures overlap conceptually with CFS grading, reduced models were fitted: omitting Katz ADL, Lawton IADL, and Mini-Cog gave an area under the curve of 0.779 (95% CI: 0.721–0.833), while additionally omitting MNA and TUG gave 0.657 (95% CI: 0.592–0.719), close to the basic clinical model. Inflammatory indices provided no consistent incremental contribution; the small increase with PLR had a bootstrap confidence interval that included zero. Conclusions: In this sample, comprehensive geriatric assessment domains demonstrated a stronger association with same-time CFS-defined frailty classification than routine blood-based inflammatory indices. The findings describe contemporaneous classification in very old, hospital-based patients and do not establish prospective predictive value.

## 1. Introduction

Frailty is a clinical syndrome characterized by reduced physiological reserve and diminished tolerance to stressors in later life [1,2]. It is not explained by chronological age or comorbidity count alone [3]. In a systematic review and meta-analysis of community-dwelling adults aged 60 years or older, the incidence of frailty was 43.4 per 1000 person-years, and the incidence of prefrailty was 150.6 per 1000 person-years, with substantial variation by diagnostic criteria, sex, and country income level [4]. These estimates describe community-dwelling populations and are not directly transferable to hospital-based older patients, among whom age, functional dependence, and comorbidity burden are typically higher. The Clinical Frailty Scale (CFS) provides a practical clinical grading approach that incorporates comorbidity, function, and cognition [5], and CFS scores have been associated with functional status, cognition, falls, and clinical outcomes [6].

Frailty often emerges from the clustering of geriatric syndromes rather than from a single deficit. Nutritional impairment, polypharmacy, multimorbidity, functional dependence, cognitive impairment, fall risk, and reduced quality of life frequently coexist and reinforce one another [7,8,9]. Therefore, models that identify which geriatric domains carry frailty may be more clinically informative than a simple frail/nonfrail classification.

Aging-related chronic, low-grade inflammation offers a plausible biological pathway for frailty [10,11,12,13]. Complete blood count and routine biochemistry-derived indices, including neutrophil-to-lymphocyte ratio (NLR), platelet-to-lymphocyte ratio (PLR), systemic immune-inflammation index (SII), C-reactive protein/albumin ratio (CAR), and inflammatory prognostic index (IPI), are attractive because they are inexpensive and readily available. Large cohort and survey-based studies have linked NLR, PLR, SII, and related inflammatory measures with frailty [14,15,16,17]. However, the extent to which these indices add clinically useful information beyond functional, cognitive, nutritional, and mobility assessment in older clinical populations remains unclear.

This study evaluated CFS-defined clinical frailty together with comprehensive geriatric assessment domains and routine blood-based inflammatory/prognostic indices in a hospital-based sample of adults aged 65 years or older who underwent comprehensive geriatric assessment at a healthy aging outpatient clinic run by a department of family medicine within a university hospital. We hypothesized that frailty would be more strongly associated with function, cognition, nutrition, and mobility than with inflammatory indices, and that NLR, PLR, SII, CAR, and IPI would provide limited incremental discrimination beyond the core geriatric model. Because geriatric assessment, CFS grading, and blood sampling were performed on the same day, all analyses address the contemporaneous classification of frailty rather than its prospective prediction.

## 2. Materials and Methods

### 2.1. Study Design and Ethics

This single-center, observational, cross-sectional study used routinely collected records of adults aged 65 years or older who underwent comprehensive geriatric assessment at the healthy aging outpatient clinic run by the Department of Family Medicine at Recep Tayyip Erdoğan University Faculty of Medicine, Rize, Türkiye. Records were identified retrospectively and consecutively from the hospital information system for the period between August 2024 and August 2025; every record meeting the eligibility criteria within that period was included, and no sampling, matching, or selection by frailty status was applied. Records included demographic characteristics, chronic diseases, medication use, comprehensive geriatric assessment findings, and routine laboratory values obtained on the same day as the clinical assessment. The study was performed in accordance with the principles of the Declaration of Helsinki (as revised in 2013). Approval was granted by the Non-Interventional Clinical Research Ethics Committee of Recep Tayyip Erdoğan University (decision number 2024/175; approval letter E-40465587-050.01.04-1139, dated 18 July 2024). All geriatric assessment instruments and laboratory tests used in this study formed part of routine clinical care at the study center; no additional intervention, contact, or biological sampling was carried out for research purposes. Written consent for clinical assessment and for the recording of clinical data was obtained from each patient, or from a relative where appropriate, at the time of the clinic visit, in accordance with standard practice at the center. No separate study-specific consent was sought, because the analysis used only these existing records, which carried no direct personal identifiers and were fully anonymized before analysis. The study is reported in accordance with the Strengthening the Reporting of Observational Studies in Epidemiology (STROBE) statement [18].

### 2.2. Analysis Population

Eligibility was assessed in two stages. At the point of record identification, patients with documented evidence of acute infection, active malignancy under treatment, systemic corticosteroid use, ongoing chemotherapy, or acute inflammatory disease at the time of assessment were not carried forward, because these conditions directly alter the blood-based indices under study. A previously treated malignancy in remission was not an exclusion criterion and was counted within the comorbidity burden. A total of 300 eligible records was screened.

At the second stage, records were checked against prespecified validity ranges. These ranges were data-quality filters rather than clinical selection criteria. For the geriatric instruments they reproduce the range of scores that each instrument can produce (1–9 for CFS, 0–6 for Katz Activities of Daily Living (ADL), 0–8 for Lawton Instrumental Activities of Daily Living (IADL), 0–14 for Mini Nutritional Assessment (MNA), 0–5 for Mini-Cog, and 0–15 for Geriatric Depression Scale-15 (GDS-15)), so that a value outside the range can only represent a transcription or data-entry error. For age (65–110 years), Timed Up and Go (TUG) time (0–60 s), and laboratory values (50–1000 × 10^3^/µL for platelets, 0.1–30 × 10^3^/µL for neutrophils, greater than 0 to 20 × 10^3^/µL for lymphocytes, 20–60 g/L for albumin, and 0–300 mg/L for C-reactive protein (CRP)), the limits were set at physiologically implausible values; the upper age limit of 110 years exceeds the oldest documented age in the national population by a wide margin and therefore flags only implausible date-of-birth entries. Records with a missing core analytic variable were also not analyzed, and no imputation was performed.

**Figure 1:**
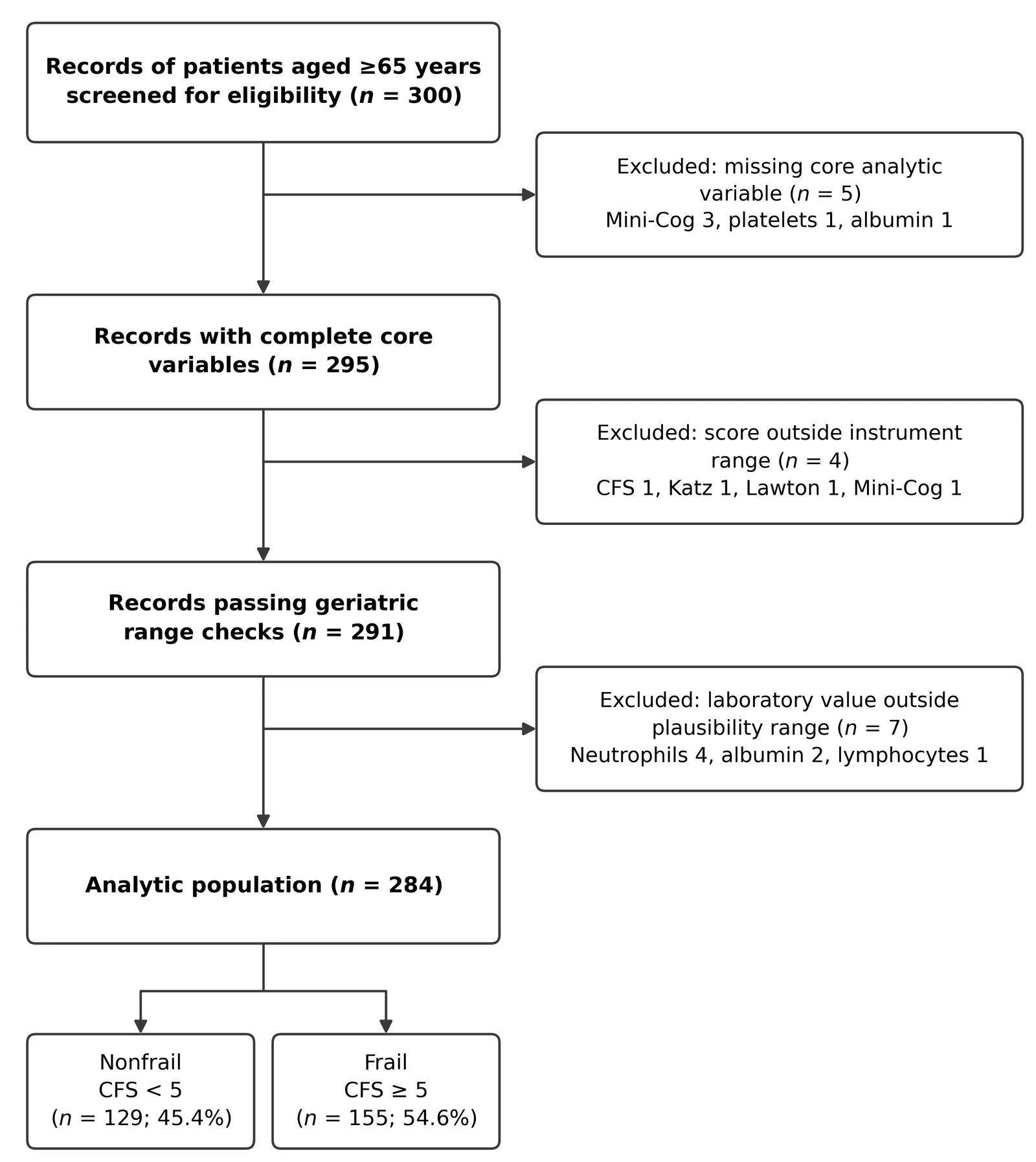
Participant flow. Records were screened for eligibility, and the clinical exclusion criteria (acute infection, active malignancy under treatment, systemic corticosteroid use, ongoing chemotherapy, and acute inflammatory disease) were applied when records were identified. The 16 exclusions shown occurred at the data-quality stage. CFS, Clinical Frailty Scale; ADL, Activities of Daily Living; IADL, Instrumental Activities of Daily Living.

Sixteen of the 300 screened records were excluded at this second stage: 5 because a core analytic variable was missing (Mini-Cog in 3, platelet count in 1, and albumin in 1), 4 because a geriatric score lay outside its instrument range (CFS in 1, Katz ADL in 1, Lawton IADL in 1, and Mini-Cog in 1), and 7 because a laboratory value lay outside the plausibility range (neutrophil count in 4, albumin in 2, and lymphocyte count in 1). No record fell outside the age range, and the single record with a TUG time above 60 s was already excluded because its Lawton IADL and Mini-Cog values were outside their instrument ranges. Because these filters removed only missing or impossible values, they are unlikely to have introduced selection bias; the one situation in which such a filter could do so, the upper limit on TUG time, is addressed in the Discussion. The participant flow is shown in Figure 1. The final analytic population consisted of 284 individuals, with no missing values for core variables.

### 2.3. Frailty Assessment and Geriatric Variables

Frailty was defined as a CFS score of 5 or higher [5]; participants with a CFS score lower than 5 were classified as nonfrail. A sensitivity analysis used a CFS score of 6 or higher as a more advanced frailty threshold. Functional status was assessed with the Katz ADL [19] and Lawton IADL [20] scores. A Katz score lower than 6 indicated ADL dependence, and a Lawton score lower than 8 indicated IADL dependence. Nutritional status was assessed by MNA [21], with a score lower than 12 indicating malnutrition risk. Cognition was assessed using the Mini-Cog [22]. Depressive symptom burden was assessed using the GDS-15 total score [23]. Mobility was assessed using TUG time [24]. Fall risk was analyzed as a binary variable recorded in the geriatric assessment form. Polypharmacy was defined as the regular use of five or more medications [25]. The cardiovascular disease (CVD) composite included hypertension, coronary artery disease, and heart failure; comorbidity count was calculated separately from this composite. The CFS is a clinical grading instrument that already incorporates comorbidity, function, and cognition; the resulting conceptual overlap with several of the measures listed below is addressed in the statistical analysis and in the Discussion.

### 2.4. Laboratory Variables and Inflammatory Indices

Inflammatory and prognostic indices were recalculated from measured laboratory values. NLR was calculated as neutrophil count divided by lymphocyte count; PLR as platelet count divided by lymphocyte count; SII as platelet count × neutrophil count/lymphocyte count [26]; CAR as CRP divided by albumin; and IPI as CRP × NLR/albumin [27]. CAR was presented as the unscaled CRP/albumin ratio.

### 2.5. Statistical Analysis

Continuous variables are reported as median and interquartile range (IQR), and categorical variables as number and percentage. Groups were compared using the Mann–Whitney U test, chi-square test, or Fisher’s exact test when expected cell counts were low. Associations between CFS score and geriatric or inflammatory variables were examined using Spearman rank correlation. Multivariable logistic regression results are presented as odds ratios (ORs), 95% confidence intervals (CIs), and *p*-values. Continuous predictors were standardized per 1-standard-deviation (SD) increase; binary predictors were entered using their original 0/1 coding. The basic clinical model included age, sex, body mass index (BMI), comorbidity count, and polypharmacy. The core geriatric model additionally included Katz ADL, Lawton IADL, MNA, TUG, GDS-15, and Mini-Cog scores. Each inflammatory index was added separately to the core geriatric model. All analyses were planned as a cross-sectional examination of the contemporaneous association between each set of measures and CFS-defined frailty classification; no longitudinal outcome was available, and the models are therefore not prediction models in the prognostic sense.

Sample size. The analysis used all eligible records identified in the study period, so no a priori sample size calculation was performed. The adequacy of the available sample was instead assessed against current recommendations for multivariable models with a binary outcome [28,29]. With 155 frail and 129 nonfrail participants and 11 parameters in the core geriatric model, there were 11.7 events per variable for the smaller outcome group. Applying the criteria of Riley and colleagues to the model actually fitted (apparent Cox–Snell R^2^ 0.365; adjusted Cox–Snell R^2^ 0.334; outcome proportion 0.546), the minimum sample required was 238 for an expected shrinkage factor of at least 0.9 and 236 for an absolute difference between apparent and adjusted Nagelkerke R^2^ of no more than 0.05 [28]. Both criteria were met, and the shrinkage factor achievable at *n* = 284 was 0.92. The corresponding criterion for estimating the overall outcome proportion within ±0.05 required 381 participants and was not met, which is reflected in the width of the confidence interval reported for frailty prevalence. The calculations are given in Table S1.

Model performance. Discrimination was assessed using receiver operating characteristic (ROC) curves and the area under the curve (AUC) with percentile bootstrap confidence intervals from 4000 resamples; the Brier score and Nagelkerke R^2^ were also calculated. Calibration was assessed by the calibration slope, the calibration-in-the-large, a loess-smoothed calibration curve, and the Hosmer–Lemeshow test [30,31]. Because every model was fitted and evaluated in the same dataset, apparent calibration is perfect by construction; optimism in the AUC, the Brier score, and the calibration slope was therefore estimated by bootstrap resampling with 1000 replications, and optimism-corrected values are reported alongside apparent values [31]. The optimism-corrected calibration slope was obtained by refitting the full model in each bootstrap resample and evaluating the calibration slope of that resample’s linear predictor in the original dataset, averaged over replications. It is therefore an empirical estimate and is not interchangeable with the analytical shrinkage factor used in the sample size assessment, which is a closed-form approximation based on the likelihood ratio statistic and is the more optimistic of the two (0.92 against 0.81; Table S1). Stratified fivefold cross-validation was used as an additional internal validation. Decision curve analysis [32] is reported in Figure S2, together with the operating characteristics of the core geriatric model at selected thresholds (Table S3).

Sensitivity analyses. Seven additional analyses were performed to examine specific sources of bias. Frailty was redefined as a CFS score of 6 or higher. Three reduced models were fitted to address the overlap between the outcome and the explanatory variables: the first omitted Katz ADL, Lawton IADL, and Mini-Cog, whose content is used directly when a CFS grade is assigned, while retaining the basic clinical variables together with MNA, TUG, and GDS-15; the second additionally omitted MNA and TUG, leaving GDS-15 as the only geriatric measure; and the third omitted Katz ADL, Lawton IADL, and TUG, that is, the functional and mobility measures most often identified as overlapping with CFS grading. Participants with a recorded history of malignancy were excluded in one analysis and participants with a CRP above 50 mg/L in another, as a check on residual acute inflammation. Finally, because the recorded differential counts were not always internally consistent with the recorded total leukocyte count, the index analyses were repeated after excluding records in which the neutrophil count exceeded the recorded total leukocyte count, and again after restricting to records in which the sum of the neutrophil and lymphocyte counts did not exceed the recorded total leukocyte count by more than 0.2 × 10^3^/µL.

A 6-item exploratory geriatric burden score was constructed as the unweighted count of ADL dependence, IADL dependence, malnutrition risk, fall risk, recorded depression history/diagnosis, and a Mini-Cog score lower than 3. The six indicators and their thresholds were specified on clinical grounds before the score was examined: each threshold is the standard published cut-off for the instrument concerned and is the same one applied elsewhere in this analysis, and no item weights, thresholds, or item selections were estimated from these data. The only quantities fitted are therefore the intercept and slope of a single-predictor logistic model, for which bootstrap resampling gave an optimism estimate below 0.001 in absolute value, within Monte Carlo error of zero; apparent performance is accordingly reported without optimism correction. The score is nevertheless presented as exploratory and hypothesis-generating, because it was evaluated in the same sample in which it was proposed and because three of its six items are among the measures whose content is used directly in CFS grading. Tests were two-sided, with *p* < 0.05 considered statistically significant. Analyses were performed using Python 3.13.5 with the pandas, SciPy, statsmodels, and scikit-learn libraries.

## 3. Results

Of the 300 screened records, 16 were excluded at the data-quality stage and 284 were analyzed (Figure 1). Of these, 155 (54.6%; 95% CI: 48.8–60.3) were frail and 129 (45.4%) were nonfrail. The median age was 84 years (IQR: 82–88), and 71 participants (25.0%) were men. Demographic, clinical, and geriatric characteristics by frailty status are shown in Table 1. Frailty groups differed most clearly by functional dependence, IADL dependence, malnutrition risk, fall risk, cognitive performance, TUG time, and GDS-15 score. By contrast, BMI, comorbidity count, CVD, multimorbidity, and polypharmacy did not differ significantly between groups.

Inflammatory and prognostic indices are summarized in Table 2. Median NLR and SII values were lower in the frail group, PLR showed a borderline between-group difference, and CAR and IPI did not differ between groups. After adjustment for basic clinical variables and core geriatric scores, NLR, SII, CAR, and IPI were not independently associated with frailty, although the estimates for NLR and SII proved sensitive to the handling of internally inconsistent leukocyte counts (Table S4). PLR showed an inverse adjusted association, but its incremental contribution to discrimination was small and is interpreted as exploratory below.

The strongest rank correlations with CFS score were observed for geriatric scales: Katz ADL (rho = −0.631), Mini-Cog (rho = −0.533), Lawton IADL (rho = −0.525), MNA (rho = −0.416), TUG time (rho = 0.402), and GDS-15 (rho = 0.273; all *p* < 0.001). NLR, PLR, SII, CAR, and IPI were not significantly correlated with CFS score. In the multivariable core model, higher Katz ADL, Lawton IADL, MNA, and Mini-Cog scores were independently associated with lower odds of frailty (Table 3). TUG showed a borderline association; age, sex, BMI, comorbidity count, polypharmacy, and GDS-15 were not independently associated with frailty after geriatric scores were entered.

**Figure 2:**
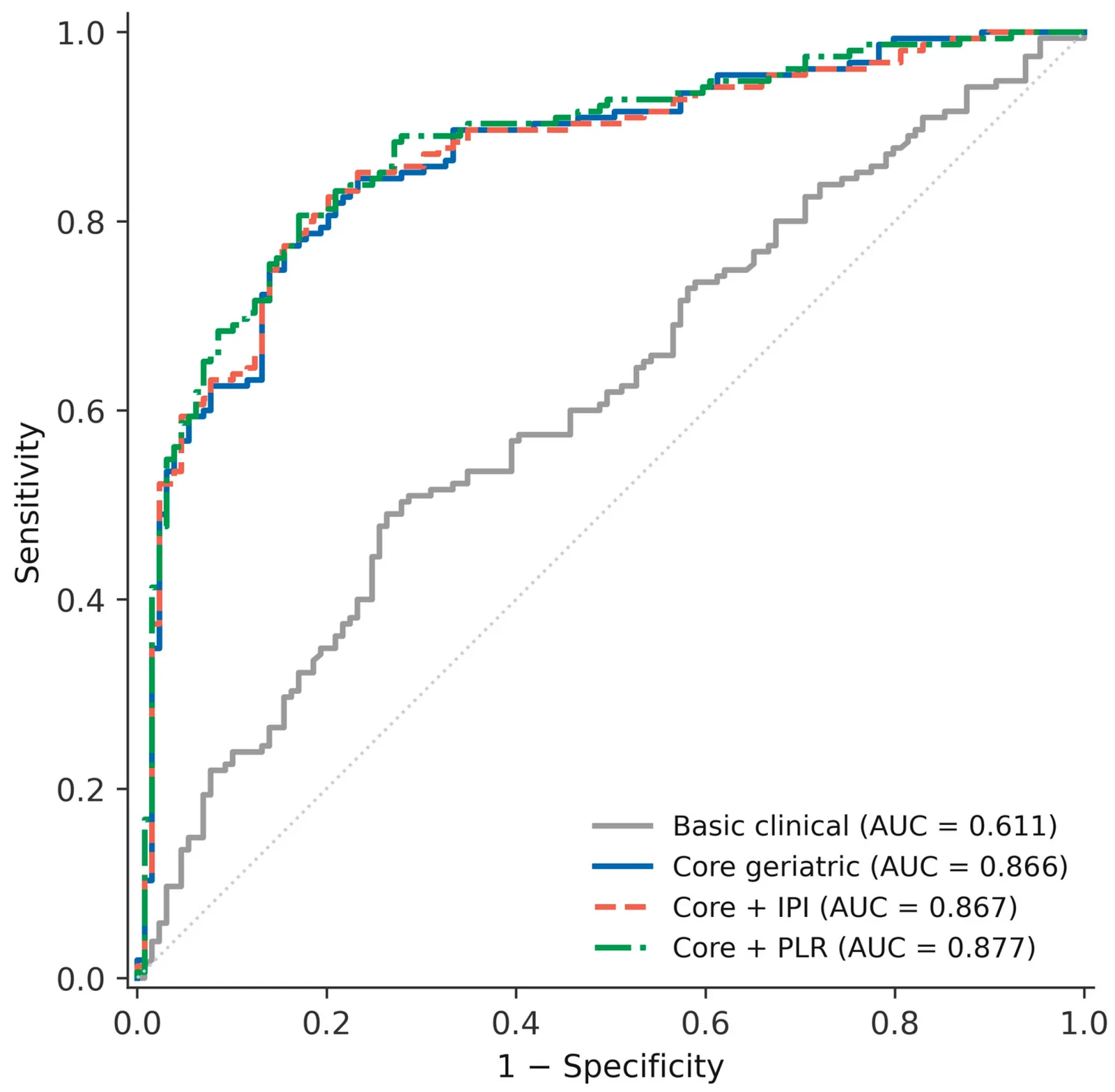
Receiver operating characteristic curves for the cross-sectional classification of frailty using basic clinical, core geriatric, and index-added models. The grey dotted diagonal line indicates chance-level discrimination (AUC = 0.5). The basic clinical model included age, sex, body mass index, comorbidity count, and polypharmacy. The core geriatric model additionally included Katz ADL, Lawton IADL, MNA, Timed Up and Go, GDS-15, and Mini-Cog. IPI and PLR were added in separate models. ADL, Activities of Daily Living; AUC, area under the curve; GDS, Geriatric Depression Scale; IADL, Instrumental Activities of Daily Living; IPI, inflammatory prognostic index; MNA, Mini Nutritional Assessment; PLR, platelet-to-lymphocyte ratio.

Model discrimination and calibration are shown in Table 4 and Figure 2. The AUC was 0.611 (95% CI: 0.545–0.677) in the basic clinical model and 0.866 (95% CI: 0.821–0.907) in the core geriatric model, a difference of 0.255 (95% bootstrap CI: 0.185–0.327). Cross-validated AUC values were 0.553 and 0.833, and bootstrap optimism-corrected values were 0.573 and 0.842, respectively. For the core geriatric model, the optimism-corrected calibration slope was 0.81, the calibration-in-the-large was 0.00, and the Hosmer–Lemeshow test was not significant (*p* = 0.06); the calibration curve is shown in Figure S1. Adding IPI did not improve the AUC. Adding PLR increased the AUC by 0.011, but the 95% bootstrap CI included zero (−0.006 to 0.029). At the threshold maximizing the Youden index, the core geriatric model classified frailty with a sensitivity of 78.1%, a specificity of 83.7%, a positive predictive value of 85.2%, and a negative predictive value of 76.1% (Table S3). Because the core geriatric model shares information with the outcome, these values describe how closely the measured domains reproduce the CFS grading rather than independent classification ability.

Sensitivity analyses are summarized in Table S2. Because several geriatric measures overlap conceptually with the information used to assign a CFS grade, two reduced models were fitted. Omitting Katz ADL, Lawton IADL, and Mini-Cog, whose content is used directly in CFS grading, gave an AUC of 0.779 (95% CI: 0.721–0.833; optimism-corrected 0.749), which remained clearly above the basic clinical model (difference 0.167; 95% CI: 0.106–0.232). Additionally omitting MNA and TUG, so that GDS-15 was the only geriatric measure retained, gave an AUC of 0.657 (95% CI: 0.592–0.719; optimism-corrected 0.623), which no longer differed clearly from the basic clinical model (difference 0.046; 95% CI: −0.005 to 0.098). A third reduced model, omitting Katz ADL, Lawton IADL, and TUG, gave an AUC of 0.802 (95% CI: 0.747–0.853), also above the basic clinical model (difference 0.191; 95% CI: 0.118–0.264). The first two reduced models are shown in Table 4 and Figure S3, and all three in Table S2. When frailty was defined as a CFS score of 6 or higher, the core geriatric model had an AUC of 0.852, the first reduced model 0.739, the second 0.630 and the third 0.782, against 0.576 for the basic clinical model (Table S2); IPI again did not improve discrimination. Excluding the 36 participants with a recorded history of malignancy (*n* = 248) left the AUC of the core geriatric model unchanged at 0.866, with an adjusted OR for PLR of 0.64 (95% CI: 0.45–0.90). Excluding the 5 participants with a CRP above 50 mg/L (*n* = 279) gave an AUC of 0.863 and an adjusted OR for PLR of 0.60 (95% CI: 0.43–0.84). No participant had a white blood cell count above 11 × 10^3^/µL. In three records the neutrophil count exceeded the recorded total leukocyte count; excluding these (*n* = 281) changed the adjusted associations for NLR (OR: 0.68; 95% CI: 0.48–0.96) and SII (OR: 0.65; 95% CI: 0.47–0.92) from null to inverse, although the corresponding increases in the AUC were 0.007 and 0.011 (Table S4). When the analysis was restricted to the 188 records whose differential counts were internally consistent with the recorded total leukocyte count, neither index was associated with frailty (NLR OR: 0.86; 95% CI: 0.57–1.30; SII OR: 0.79; 95% CI: 0.53–1.18) and neither improved discrimination. The inconsistency was not independent of frailty status: it affected 65 of 155 frail participants (41.9%) and 31 of 129 nonfrail participants (24.0%) (*p* = 0.002), and all three records in which the neutrophil count exceeded the recorded total were frail. No index improved the AUC by more than 0.013 in any of these analyses.

**Figure 3:**
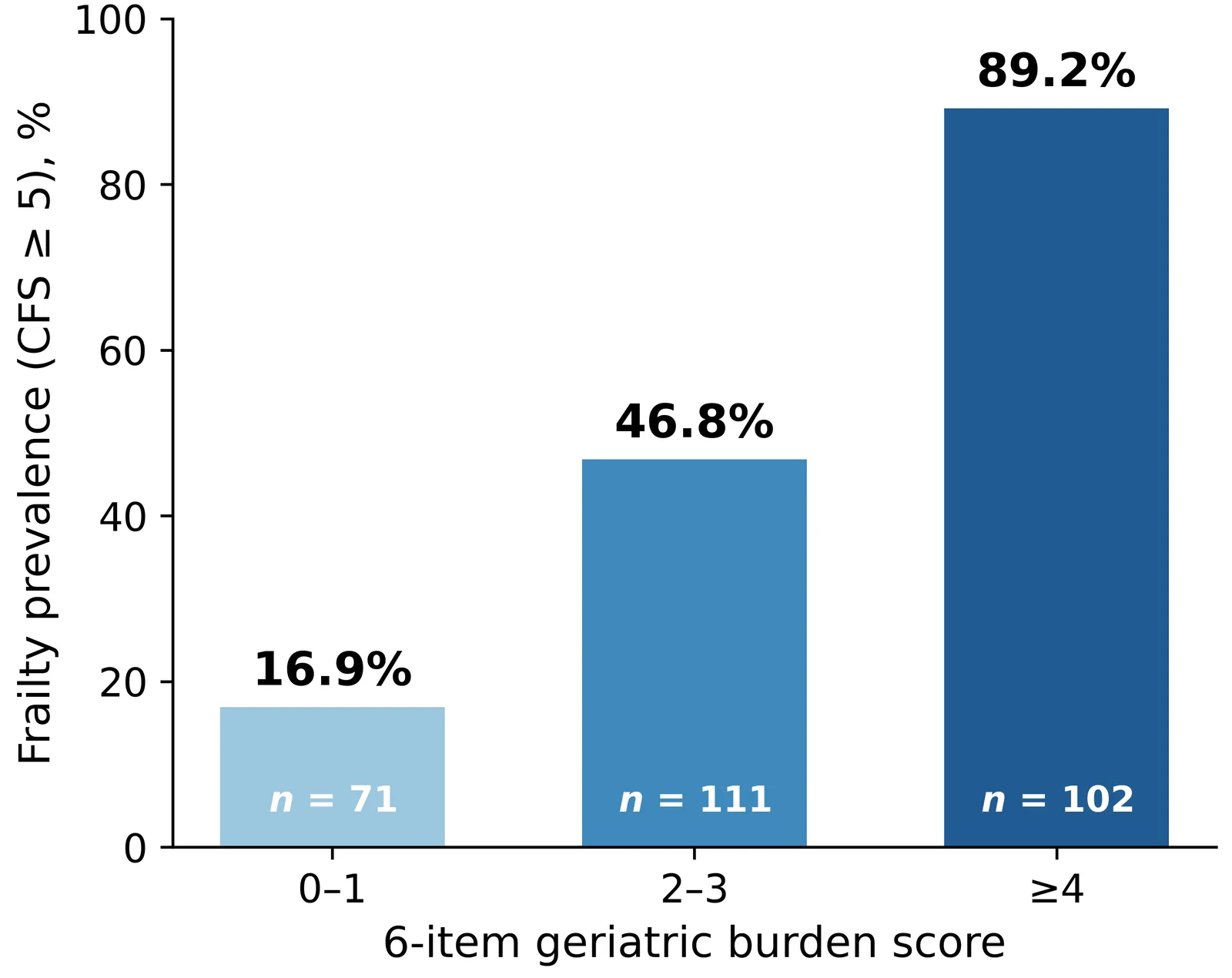
Frailty prevalence according to the exploratory 6-item geriatric burden score. Bars represent the proportion of participants with a CFS score ≥ 5 according to score level; values above the bars indicate percentages. The score ranged from 0 to 6, was derived and evaluated in the same dataset, and was not externally validated. CFS, Clinical Frailty Scale.

The exploratory 6-item geriatric burden score showed a graded relationship with frailty (Figure 3). Frailty prevalence was 16.9% among those with scores of 0–1, 46.8% among those with scores of 2–3, and 89.2% among those with scores of 4 or higher. Each 1-point increase was associated with frailty (OR: 2.65; 95% CI: 2.11–3.33; *p* < 0.001), and the score alone had an AUC of 0.839 (95% CI: 0.792–0.882). Because the items and their thresholds were specified in advance and no weights were estimated, bootstrap optimism was negligible (below 0.001 in absolute value) and apparent performance is reported. Three of the six items are measures whose content is used directly in CFS grading, so the overlap described above applies to the score as well; a reduced score built only from malnutrition risk, fall risk, and recorded depression history/diagnosis had an AUC of 0.687 (95% CI: 0.629–0.743). These values describe the behavior of the score in the present sample and should not be read as validated performance.

## 4. Discussion

In this cross-sectional study of hospital-based older adults, CFS-defined frailty was most strongly associated with functional dependence, cognitive performance, nutritional status, and mobility. The core geriatric model classified same-time frailty status considerably better than a basic model based on age, sex, BMI, comorbidity burden, and polypharmacy. Routine inflammatory/prognostic indices did not add consistent information beyond the geriatric domains. Because geriatric assessment, CFS grading, and blood sampling were contemporaneous, these results describe association and classification at a single time point and do not support statements about prospective risk prediction.

The observed frailty prevalence of 54.6% (95% CI: 48.8–60.3) was higher than estimates from community-based samples. A systematic review and meta-analysis of community-dwelling older adults in Asian countries reported an overall frailty prevalence of 20.5% and a prevalence of 34.0% among those aged 85 years or older, with substantial variation by assessment tool [33]. Several features of the present study make this comparison indirect. The setting differs because the present records came from patients referred for comprehensive geriatric assessment at a university hospital, whereas the meta-analysis pooled community surveys. The age distribution differs, because the median age here was 84 years, close to the oldest stratum of the pooled estimate. The sampling differs, because consecutive clinical records were used rather than population sampling. The instrument and the threshold also differ, because the CFS incorporates comorbidity, function, and cognition and was applied with a cut-off of 5 or higher, whereas many of the pooled studies used the Fried phenotype, which weights physical performance and unintentional weight loss and tends to classify fewer people as frail in the same population. Health system context contributes as well, since referral pathways concentrate functionally impaired patients in hospital-based assessment records. Estimates from other clinical settings are closer to the present one, although none is directly comparable; in an acute inpatient surgical cohort graded with the CFS, 51.9% of patients had a score of 4 or higher [34], a figure obtained in a different care setting and at a lower threshold than the one applied here. The CFS version 2.0 has been validated for use in Turkish outpatients, which supports its applicability in this setting [35].

Functional and cognitive domains formed the central axis of frailty classification in this analysis. Similar associations between frailty, limitations in activities of daily living, cognitive impairment, and falls have been reported among community-dwelling older adults in China [36]. Acute-care data have also shown that higher CFS-defined frailty is accompanied by greater detection of geriatric syndromes, including cognitive impairment, fall risk, and malnutrition risk [34]. Nutritional status was also relevant in the present study, consistent with evidence that malnutrition and frailty frequently coexist among older adults in long-term care settings [37].

These associations must be read against the conceptual overlap between the outcome and several of the explanatory variables. The CFS is assigned by clinical judgment that already takes function and cognition into account, so Katz ADL, Lawton IADL, and Mini-Cog partly reproduce information contained in the grading itself. The high area under the curve of the core geriatric model therefore reflects construct convergence as well as association, and it should not be read as independent discriminative performance. We quantified this with two reduced models. Removing the three measures whose content is used directly in CFS grading lowered discrimination from 0.866 to 0.779, a difference of 0.088 (95% CI: 0.036–0.140), and the reduced model still separated frail from nonfrail participants clearly better than the basic clinical model (0.611; difference 0.167; 95% CI: 0.106–0.232). Removing MNA and TUG as well lowered it to 0.657, which no longer differed clearly from the basic clinical model (difference 0.046; 95% CI: −0.005 to 0.098). Nutritional status and timed mobility are measured rather than graded and are not items of the CFS, but they are closely related to the domains a clinician weighs when assigning a grade. The short form of the MNA (MNA-SF) in particular contains a mobility item and an item on neuropsychological problems, so it is not a purely nutritional measure and shares content with CFS grading more directly than its name suggests. A third reduced model, omitting Katz ADL, Lawton IADL, and TUG, gave an intermediate area under the curve of 0.802 (95% CI: 0.747–0.853). Taken together, these analyses indicate that construct overlap accounts for a substantial part of the apparent advantage of geriatric assessment over age, sex, BMI, comorbidity count, and polypharmacy, that a clear advantage nevertheless remains once the directly graded components are removed, and that the discrimination of the full core geriatric model should be treated as an upper bound rather than an estimate of independent classification ability. A stronger test would compare CFS-defined frailty with a reference measure that shares none of its components, such as the Fried phenotype; grip strength, gait speed, exhaustion, and unintentional weight loss were not recorded in these routine records, so such an analysis was not possible here and remains a priority for prospective work.

Inflammation-related findings were less consistent. Chronic low-grade inflammation is biologically plausible in frailty, and studies have associated inflammatory biomarkers with frailty phenotypes or frailty indices [11,12,13,38]. Several large cohort and survey-based analyses have reported positive associations of NLR, PLR, SII, and related blood cell-derived markers with frailty or incident frailty [14,15,16,17,39]. More recent geriatric data have also suggested that inflammatory markers may contribute to frailty risk when combined with clinical variables [40]. In the present study, however, these indices lost explanatory strength after functional, cognitive, nutritional, and mobility measures were included. The estimates for NLR and SII were moreover not stable: they changed from null to inverse when the three records whose neutrophil count exceeded the recorded total leukocyte count were removed, and returned to null when the analysis was restricted to records with an internally consistent differential. That the removal of three records moves the adjusted odds ratio for NLR from 1.00 to 0.68 shows that these estimates rest on a small number of influential extreme values. The inconsistency is also differential with respect to the outcome, since it was almost twice as frequent among frail as among nonfrail participants and all three impossible records were frail. Measurement error of this kind cannot be assumed to bias estimates towards the null, and its direction here cannot be determined from the available data; the null findings for NLR and SII should therefore be read as uninformative about any underlying association rather than as evidence against one. Across all of these analyses no index improved discrimination by more than 0.013. Differences in frailty definition, clinical setting, exclusion of acute inflammatory conditions, and reliance on a single same-day laboratory measurement may explain this discrepancy. Similar caution is supported by data from older patients hospitalized with coronavirus disease 2019, in whom CFS-defined frailty was not uniformly paralleled by higher routine inflammatory indices [41].

The inverse adjusted association observed for PLR should be regarded as exploratory rather than as a biological finding. It may reflect the distribution of blood count components in this clinical sample, residual confounding by conditions or medications that affect platelet and lymphocyte counts, biological and day-to-day variation in a single measurement, or chance arising from the separate testing of five indices. The increase in the area under the curve was 0.011 with a bootstrap confidence interval that included zero. The estimate was essentially unchanged when participants with a recorded malignancy history were excluded (OR: 0.64; 95% CI: 0.45–0.90), but it was no longer significant when frailty was defined as a CFS score of 6 or higher (OR: 0.79; 95% CI: 0.58–1.09) and was only borderline among the records whose differential counts were internally consistent (OR: 0.66; 95% CI: 0.44–1.00). PLR should therefore not be used alone as a clinical frailty marker.

The study has several strengths. It evaluated functional, cognitive, nutritional, psychological, and mobility domains within the same analytic framework; laboratory values were obtained on the same day as the geriatric assessment; major acute inflammatory conditions were excluded; and inflammatory indices were recalculated from measured values. The comparison of basic clinical, core geriatric, and index-added models allowed direct assessment of the incremental contribution of routine inflammatory measures, and the additional model without the CFS-overlapping domains quantifies how much of that contribution survives the removal of construct overlap. The assessments were also performed by family physicians in a family medicine-run clinic rather than in a specialist geriatrics department, so the measures used here are those available to the discipline for which the findings are most relevant. For primary care and family medicine, the findings are consistent with a patient-centered and multidimensional approach to frailty recognition, in which simple assessments of function, nutrition, cognition, and mobility may be more informative than laboratory indices alone [42,43]. These strengths do not imply generalizability: the analytic population had a median age of 84 years and was drawn from a hospital-based assessment service, so the findings describe very old patients already referred for geriatric assessment rather than the wider population of adults aged 65 years or older.

This study has several limitations. The cross-sectional design precludes causal inference and permits no statement about prospective prediction; the models describe classification at a single time point. The single-center population, drawn from a hospital-based healthy aging clinic and with a median age of 84 years, limits generalizability to younger, healthier, community-dwelling older adults, and the frailty prevalence reported here should not be read as a population estimate. The available sample met current criteria for the degree of overfitting expected in the core geriatric model but fell short of the sample size required to estimate the overall frailty proportion within ±0.05. Several model components overlap conceptually with CFS grading. The reduced models show that this overlap accounts for a substantial part of the model’s performance: a clear advantage over the basic clinical variables remained once the three directly graded components were removed, but it did not survive the further removal of the Mini Nutritional Assessment and Timed Up and Go. The discrimination of the full core geriatric model is therefore an upper bound rather than an estimate of independent classification ability. No external validation was possible, and the optimism-corrected calibration slope of 0.81 indicates that the fitted coefficients would require shrinkage before any use elsewhere. Inflammatory indices were based on one routine blood sample, and more detailed immune markers, such as interleukin-6, tumor necrosis factor-alpha, and monocyte-based indices, were not available. Acute infection and acute inflammatory disease were excluded on the basis of the documented clinical assessment rather than a laboratory threshold, although no participant had leukocytosis and only five had a CRP above 50 mg/L. The clinical exclusion criteria were applied when records were identified, as part of routine eligibility screening, and the number of records excluded under each criterion was not retained; we are therefore unable to report these counts. The recorded differential counts were not always internally consistent with the recorded total leukocyte count, which affected the estimates for NLR and SII as described above; the indices were recalculated from the recorded values, and no retrospective correction was possible. Because the inconsistency was substantially more frequent among frail participants, the resulting measurement error is differential and its effect on the index estimates cannot be signed. Fall risk was recorded as a binary clinical variable rather than by a standardized scale. Recorded depression history/diagnosis was a clinical record variable and not a diagnosis derived from GDS-15. The upper limit of 60 s applied to TUG time is a data-quality filter that could in principle exclude the slowest walkers; one record exceeded it and was also excluded for other reasons. The exploratory geriatric burden score was derived and evaluated in the same sample and requires external validation before any clinical use.

## 5. Conclusions

In this sample of hospital-based older adults, comprehensive geriatric assessment domains demonstrated a stronger association with same-time CFS-defined frailty classification than routine blood-based inflammatory indices. A substantial part of this advantage reflects the conceptual overlap between the CFS and the functional and cognitive measures it incorporates, although a clear advantage over basic clinical variables remained after the directly graded components were removed; when the Mini Nutritional Assessment and Timed Up and Go were removed as well, that advantage was no longer clear. Routine laboratory indices added no measurable information beyond the geriatric domains, and clinical decision-making should remain grounded in comprehensive geriatric assessment. Multicenter prospective studies with external validation are needed to determine whether inflammatory indices add value for predicting longitudinal outcomes and to evaluate practical multidimensional frailty screening models for primary care.

## Figures and Tables

**Table 1:** Demographic, clinical, and geriatric characteristics according to frailty status.

Variable	Total (*n* = 284)	Nonfrail (CFS < 5; *n* = 129)	Frail (CFS ≥ 5; *n* = 155)	*p*-Value
Age, years, median (IQR)	84 (82–88)	84 (82–86)	85 (82–89)	0.020
Male sex, *n* (%)	71 (25.0)	40 (31.0)	31 (20.0)	0.033
Body mass index, kg/m^2^, median (IQR)	30.7 (27.0–33.5)	30.0 (27.0–33.0)	31.0 (26.2–34.0)	0.680
Number of comorbidities (excluding CVD), median (IQR)	4 (3–5)	4 (3–5)	4 (3–5)	0.927
Cardiovascular disease, *n* (%)	274 (96.5)	122 (94.6)	152 (98.1)	0.194
Multimorbidity ≥ 3, *n* (%)	243 (85.6)	112 (86.8)	131 (84.5)	0.582
Polypharmacy, *n* (%)	122 (43.0)	60 (46.5)	62 (40.0)	0.270
CFS score, median (IQR)	5 (4–6)	3 (3–4)	6 (5–7)	—
Katz ADL score, median (IQR)	6 (5–6)	6 (6–6)	5 (4–6)	<0.001
ADL dependence (Katz < 6), *n* (%)	92 (32.4)	5 (3.9)	87 (56.1)	<0.001
Lawton IADL score, median (IQR)	8 (5–8)	8 (8–8)	6 (4–8)	<0.001
IADL dependence (Lawton < 8), *n* (%)	134 (47.2)	27 (20.9)	107 (69.0)	<0.001
MNA score, median (IQR)	13 (12–13)	13 (12–13)	12 (11–13)	<0.001
Malnutrition risk (MNA < 12), *n* (%)	60 (21.1)	12 (9.3)	48 (31.0)	<0.001
Timed Up and Go, s, median (IQR)	13 (11–18)	12 (10–15)	15 (12–19)	<0.001
Fall risk, *n* (%)	201 (70.8)	68 (52.7)	133 (85.8)	<0.001
GDS-15 score, median (IQR)	3 (2–5)	3 (2–4)	4 (2–6)	0.001
Recorded depression history/diagnosis, *n* (%)	144 (50.7)	60 (46.5)	84 (54.2)	0.197
Mini-Cog score, median (IQR)	2 (1–3)	3 (2–3)	1 (0–2)	<0.001
Incontinence, *n* (%)	16 (5.6)	11 (8.5)	5 (3.2)	0.054

Continuous variables are presented as median (interquartile range) and categorical variables as number (percentage); the applicable format is stated in each row label. ADL dependence was defined as a Katz score < 6, IADL dependence as a Lawton score < 8, and malnutrition risk as an MNA score < 12. Polypharmacy was defined as the regular use of five or more medications. The CVD composite included hypertension, coronary artery disease, and heart failure. Recorded depression history/diagnosis refers to a binary clinical record variable and was not derived from the GDS score. The CFS score was the grouping variable; therefore, no *p*-value is provided for that row. ADL, Activities of Daily Living; CFS, Clinical Frailty Scale; CVD, cardiovascular disease; GDS, Geriatric Depression Scale; IADL, Instrumental Activities of Daily Living; IQR, interquartile range; MNA, Mini Nutritional Assessment.

**Table 2:** Distribution of inflammatory and prognostic indices and adjusted associations.

Index	Nonfrail (CFS < 5)	Frail (CFS ≥ 5)	Between-Group *p*	Adjusted OR (95% CI)	Adjusted *p*
NLR	1.70 (1.24–2.23)	1.52 (1.03–1.95)	0.017	1.00 (0.77–1.31)	0.976
PLR	96.00 (67.74–133.14)	85.56 (62.50–116.25)	0.055	0.60 (0.43–0.84)	0.003
SII	360.0 (238.5–505.9)	284.5 (211.1–424.2)	0.029	0.89 (0.65–1.22)	0.470
CAR (CRP/albumin)	0.07 (0.04–0.20)	0.10 (0.05–0.20)	0.424	1.22 (0.87–1.72)	0.252
IPI	0.14 (0.06–0.38)	0.14 (0.05–0.30)	0.756	1.19 (0.87–1.62)	0.271

Data are presented as median (IQR). Adjusted ORs were calculated for a 1-SD increase in each index added separately to the model containing basic clinical variables and Katz ADL, Lawton IADL, MNA, TUG, GDS, and Mini-Cog scores. CAR, C-reactive protein/albumin ratio; CI, confidence interval; IPI, inflammatory prognostic index; IQR, interquartile range; NLR, neutrophil-to-lymphocyte ratio; OR, odds ratio; PLR, platelet-to-lymphocyte ratio; SII, systemic immune-inflammation index.

**Table 3:** Multivariable core model associated with frailty.

Variable	Modeling Unit	Adjusted OR (95% CI)	*p*-Value
Age	Per 1-SD increase	1.12 (0.82–1.54)	0.480
Male sex	1 vs. 0	1.13 (0.55–2.31)	0.746
Body mass index	Per 1-SD increase	0.98 (0.71–1.37)	0.924
Number of comorbidities (excluding CVD)	Per 1-SD increase	0.92 (0.66–1.27)	0.610
Polypharmacy	1 vs. 0	1.01 (0.54–1.88)	0.982
Katz ADL score	Per 1-SD increase	0.52 (0.28–0.97)	0.039
Lawton IADL score	Per 1-SD increase	0.44 (0.29–0.67)	<0.001
MNA score	Per 1-SD increase	0.59 (0.36–0.95)	0.029
Timed Up and Go	Per 1-SD increase	1.41 (1.00–2.00)	0.052
GDS-15 score	Per 1-SD increase	0.99 (0.69–1.44)	0.972
Mini-Cog score	Per 1-SD increase	0.52 (0.37–0.74)	<0.001

The dependent variable was frailty defined as a CFS score ≥ 5. For continuous variables, ORs correspond to a 1-SD increase. The model is a cross-sectional discrimination model for same-time CFS classification, not a prospective prediction tool. CI, confidence interval; OR, odds ratio; SD, standard deviation.

**Table 4:** Model discrimination, internal validation, and calibration.

Model	AUC (95% CI)	Internal Validation AUC ^a^	Calibration Slope ^b^	Nagelkerke R^2^	Brier Score	ΔAUC (95% CI) ^c^
Basic clinical model	0.611 (0.545–0.677)	0.573; 0.553	0.71	0.047	0.239	—
Core geriatric model	0.866 (0.821–0.907)	0.842; 0.833	0.81	0.488	0.145	0.255 (0.185 to 0.327)
Core geriatric + IPI	0.867 (0.821–0.908)	0.840; 0.832	0.80	0.492	0.144	0.000 (−0.005 to 0.006)
Core geriatric + PLR	0.877 (0.834–0.917)	0.852; 0.843	0.80	0.518	0.138	0.011 (−0.006 to 0.029)
Core geriatric model without Katz ADL, Lawton IADL, and Mini-Cog	0.779 (0.721–0.833)	0.749; 0.735	0.81	0.229	0.197	−0.088 (−0.140 to −0.036)
Core geriatric model without Katz ADL, Lawton IADL, Mini-Cog, MNA, and TUG	0.657 (0.592–0.719)	0.623; 0.605	0.79	0.093	0.230	−0.209 (−0.275 to −0.145)

ΔAUC was calculated relative to the basic clinical model for the core geriatric model, and relative to the core geriatric model for the index-added and reduced models. Confidence intervals for the AUC and for ΔAUC were obtained by percentile bootstrap with 4000 resamples. ^a^ Internal validation AUC is reported as the bootstrap optimism-corrected value (1000 replications), followed by the value from stratified fivefold cross-validation. ^b^ Calibration slopes are optimism-corrected; the apparent slope is 1.00 for every model because each model was fitted and evaluated in the same dataset. ^c^ ΔAUC values were computed from unrounded estimates and therefore may differ slightly from the difference between the rounded AUCs shown. The reduced models retained age, sex, body mass index, comorbidity count, polypharmacy, and GDS-15, together with MNA and TUG in the first of the two. AUC, area under the curve; CI, confidence interval; GDS, Geriatric Depression Scale; IPI, inflammatory prognostic index; MNA, Mini Nutritional Assessment; PLR, platelet-to-lymphocyte ratio; TUG, Timed Up and Go.

## Data Availability

The data presented in this study are available on reasonable request from the corresponding author. The data are not publicly available due to institutional data-protection restrictions on patient records.

## Supplementary Materials

The following supporting information can be downloaded at: https://www.mdpi.com/article/10.3390/healthcare14203375/s1, Table S1: Post hoc assessment of sample size adequacy for the core geriatric model; Table S2: Sensitivity analyses; Table S3: Operating characteristics of the core geriatric model at selected thresholds; Table S4: Adjusted associations and incremental discrimination of the inflammatory indices under different handling of internally inconsistent leukocyte counts; Figure S1: Calibration of the core geriatric model; Figure S2: Decision curve analysis; Figure S3: Discrimination of the core geriatric model and of two reduced models.
