# Supplementary material for: Comprehensive Geriatric Assessment Domains and Routine Inflammatory Indices in Relation to Clinical Frailty Scale-Defined Frailty: A Cross-Sectional Study in Hospital-Based Older Adults
Source: Healthcare (Basel). 2026 Oct 10;14(20):3375. doi: 10.3390/healthcare14203375 (PMC13704348; doi:10.3390/healthcare14203375)
Supplement: Supplementary file 1 [file healthcare-14-03375-s001.zip › healthcare-4554991-supplementary.pdf]

Supplementary Material

Comprehensive Geriatric Assessment Domains and Routine Inflammatory Indices in Relation to Clinical Frailty Scale-Defined Frailty: A Cross-Sectional Study in Hospital-Based Older Adults

Latife Merve Yıldız, Handan Duman and Damla Tüfekçi

Table S1. Post hoc assessment of sample size adequacy for the core geriatric model.

Criterion	Requirement	Required n	Available n	Met
Events per variable	≥10 events per model parameter in the smaller outcome group (11 parameters, 110 events needed)	243	284 (129 events; 11.7 per parameter)	Yes
Riley criterion 1	Expected shrinkage factor ≥0.90	238	284 (shrinkage 0.92)	Yes
Riley criterion 2	Absolute difference between apparent and adjusted Nagelkerke R^2 ≤0.05	236	284	Yes
Riley criterion 3	Overall outcome proportion estimated within ±0.05	381	284	No

Values used in the calculations: 11 model parameters; outcome proportion 0.546; apparent Cox–Snell R^2 0.365; Van Houwelingen shrinkage 0.915; adjusted Cox–Snell R^2 0.334; maximum attainable Cox–Snell R^2 0.748; Nagelkerke R^2 0.488. The criteria and formulae are those of Riley and colleagues; the events-per-variable rule follows Peduzzi and colleagues. Criterion 3 concerns the precision of the estimated overall frailty proportion rather than the amount of overfitting in the model, and its shortfall is reflected in the 95% confidence interval reported for frailty prevalence (48.8–60.3%). The Van Houwelingen shrinkage factor of 0.915 shown here is a closed-form approximation derived from the likelihood ratio statistic. It is not the same quantity as the bootstrap calibration slope of 0.81 reported in Table 4, which is obtained by refitting the model in each resample and evaluating the calibration slope of that resample's linear predictor in the original data. The analytical approximation is the more optimistic of the two, and the bootstrap estimate is the one used when judging how much shrinkage the model would require.

Table S2. Sensitivity analyses.

Analysis	n	Frail, n (%)	Core geriatric model AUC	Adjusted OR for PLR (95% CI)
Main analysis (CFS ≥5)	284	155 (54.6)	0.866 (0.821–0.907)	0.60 (0.43–0.84)
Frailty defined as CFS ≥6	284	103 (36.3)	0.852	0.79 (0.58–1.09)
CFS ≥6, without Katz ADL, Lawton IADL, and Mini-Cog [a]	284	103 (36.3)	0.739 (0.680–0.795)	Not applicable
CFS ≥6, also without MNA and TUG [a]	284	103 (36.3)	0.630 (0.562–0.695)	Not applicable
CFS ≥6, without Katz ADL, Lawton IADL, and TUG [a]	284	103 (36.3)	0.782 (0.725–0.836)	Not applicable
CFS ≥6, basic clinical model	284	103 (36.3)	0.576 (0.504–0.644)	Not applicable
Without Katz ADL, Lawton IADL, and Mini-Cog [a]	284	155 (54.6)	0.779 (0.721–0.833)	Not applicable
Also without MNA and TUG [a]	284	155 (54.6)	0.657 (0.592–0.719)	Not applicable
Without Katz ADL, Lawton IADL, and TUG [a]	284	155 (54.6)	0.802 (0.747–0.853)	Not applicable
Excluding recorded malignancy history	248	137 (55.2)	0.866	0.64 (0.45–0.90)
Excluding CRP >50 mg/L	279	152 (54.5)	0.863	0.60 (0.43–0.84)
Excluding both	243	134 (55.1)	0.864	0.64 (0.46–0.91)
Excluding records with neutrophils exceeding the recorded total leukocyte count	281	152 (54.1)	0.867	0.59 (0.42–0.83)

Restricted to internally consistent differential [b]	188	90 (47.9)	0.882	0.66 (0.44–1.00)

[a] All three reduced models retain age, sex, body mass index, comorbidity count, polypharmacy, and GDS-15; the first additionally retains MNA and TUG, and the third retains MNA and Mini-Cog. [b] Records in which the sum of the neutrophil and lymphocyte counts did not exceed the recorded total leukocyte count by more than 0.2 $\times 10^3/\mu L$. Odds ratios for PLR correspond to a 1-SD increase in the index added to the core geriatric model. AUC, area under the curve; CFS, Clinical Frailty Scale; CI, confidence interval; CRP, C-reactive protein; MNA, Mini Nutritional Assessment; OR, odds ratio; PLR, platelet-to-lymphocyte ratio; TUG, Timed Up and Go. The internally inconsistent records were not evenly distributed between the outcome groups: they comprised 65 of 155 frail participants (41.9%) and 31 of 129 nonfrail participants (24.0%) (chi-square p = 0.002), and all three records in which the neutrophil count exceeded the recorded total leukocyte count were frail.

Table S3. Operating characteristics of the core geriatric model at selected thresholds.

Threshold (predicted probability)	Sensitivity, %	Specificity, %	PPV, %	NPV, %	Accuracy, %
0.500	78.1	81.4	83.4	75.5	79.6
0.538 (maximum Youden index) [a]	78.1	83.7	85.2	76.1	80.6
0.600	72.3	86.0	86.2	72.1	78.5
0.700	61.3	92.2	90.5	66.5	75.4

[a] The exact threshold maximizing sensitivity + specificity − 1 was 0.5376. These values describe how closely the measured geriatric domains reproduce the contemporaneous CFS grading; because the model shares information with the outcome, they are not estimates of screening performance against an independent reference standard. NPV, negative predictive value; PPV, positive predictive value.

Table S4. Adjusted associations and incremental discrimination of the inflammatory indices under different handling of internally inconsistent leukocyte counts.

Index	Full sample (n = 284) OR (95% CI)	ΔAUC	Excluding neutrophils > total leukocyte count (n = 281) OR (95% CI)	ΔAUC	Consistent differential (n = 188) OR (95% CI)	ΔAUC
NLR	1.00 (0.77–1.31)	0.000	0.68 (0.48–0.96)	0.007	0.86 (0.57–1.30)	−0.001
PLR	0.60 (0.43–0.84)	0.011	0.59 (0.42–0.83)	0.013	0.66 (0.44–1.00)	0.006
SII	0.89 (0.65–1.22)	0.000	0.65 (0.47–0.92)	0.011	0.79 (0.53–1.18)	0.001
CAR	1.22 (0.87–1.72)	0.001	1.23 (0.87–1.74)	0.001	1.19 (0.73–1.94)	−0.001
IPI	1.19 (0.87–1.62)	0.000	1.18 (0.87–1.60)	0.000	1.15 (0.75–1.75)	−0.002

Each index was added separately to the core geriatric model and is expressed per 1-SD increase. ΔAUC is the change in the area under the curve relative to the core geriatric model fitted in the same set of records. The adjusted associations for NLR and SII were not stable across these definitions, whereas none of the indices changed discrimination by more than 0.013 in any of them. CAR, C-reactive protein/albumin ratio; IPI, inflammatory prognostic index; NLR, neutrophil-to-lymphocyte ratio; PLR, platelet-to-lymphocyte ratio; SII, systemic immune-inflammation index.

Figure S1. Calibration of the core geriatric model.

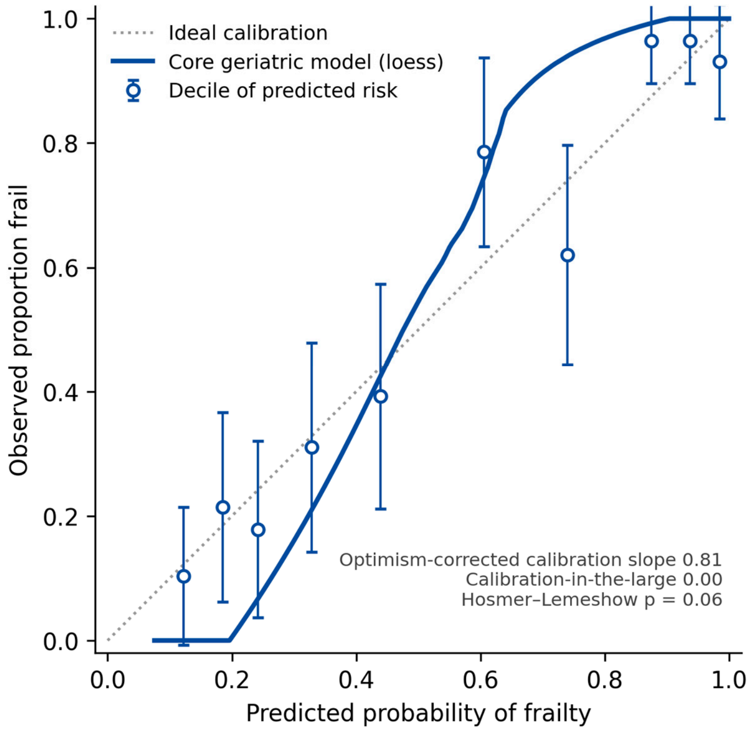

Observed proportion frail plotted against the probability predicted by the core geriatric model. Circles show deciles of predicted risk with 95% confidence intervals; the solid line is a loess smoother. Because the model was fitted and evaluated in the same dataset, the apparent calibration slope is 1.00 by construction; the bootstrap optimism-corrected slope was 0.81, indicating that the coefficients would require shrinkage before use in another sample. The calibration-in-the-large was 0.00 and the Hosmer–Lemeshow test was not significant ($\chi^2 = 15.11$, df = 8, p = 0.06).

Figure S2. Decision curve analysis.

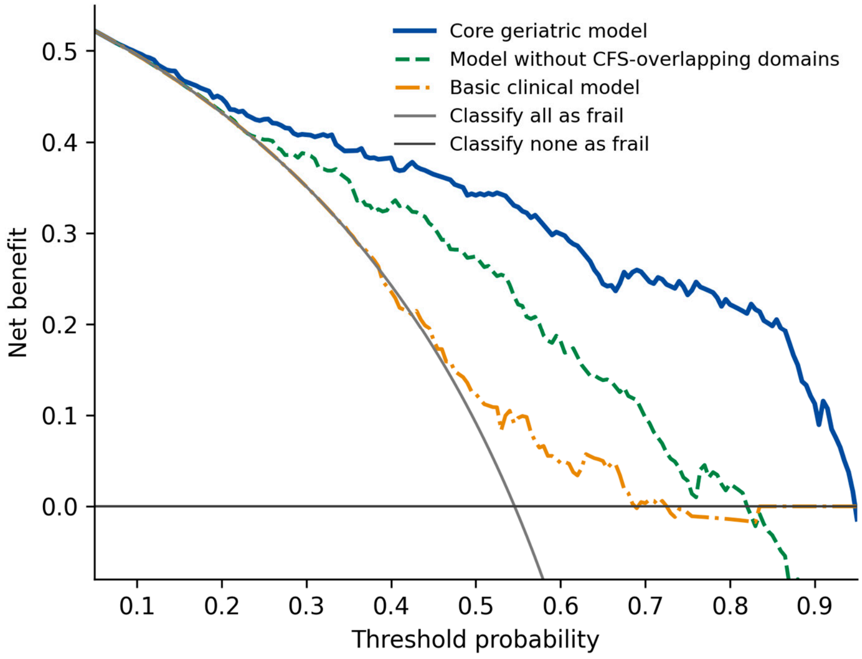

Net benefit of the core geriatric model, of the model without the CFS-overlapping domains, and of the basic clinical model, compared to classifying all or no participants as frail. The core geriatric model gave the highest net benefit across threshold probabilities from about 0.15 to 0.90. This analysis is presented for completeness in response to peer review and should be read with an important caveat: the predictors are themselves components of comprehensive geriatric assessment and the outcome was recorded at the same visit, so the curve does not describe a real decision problem in which the model would be used to decide whether to perform a geriatric assessment.

Figure S3. Discrimination of the core geriatric model and of two reduced models.

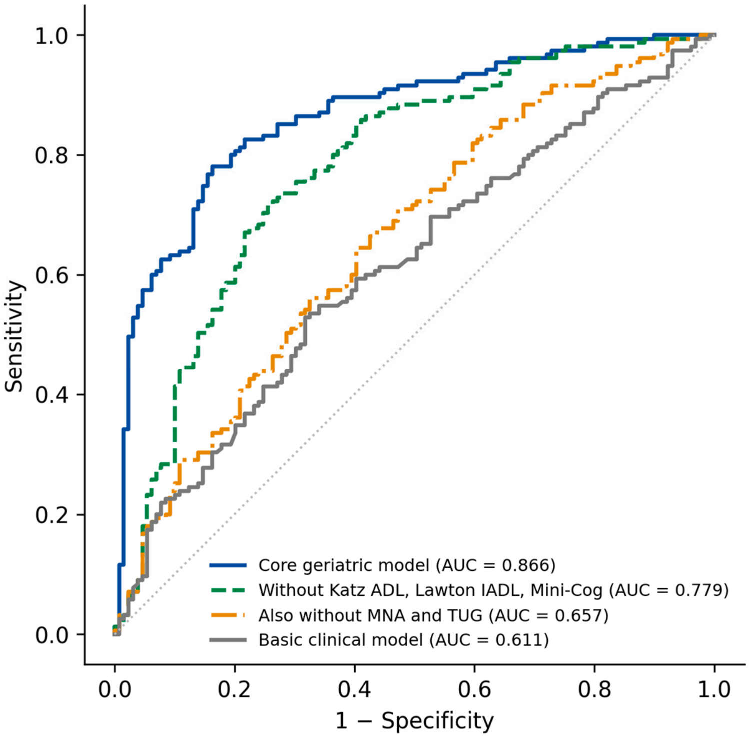

Receiver operating characteristic curves for the core geriatric model, for two reduced models, and for the basic clinical model. Removing Katz ADL, Lawton IADL, and Mini-Cog, whose content is used directly in CFS grading, reduced the area under the curve from 0.866 to 0.779 (difference 0.088; 95% CI: 0.036–0.140; optimism-corrected 0.749), which remained clearly above the basic clinical model (0.611; difference 0.167; 95% CI: 0.106–0.232). Additionally removing MNA and TUG reduced it to 0.657, which no longer differed clearly from the basic clinical model (difference 0.046; 95% CI: −0.005 to 0.098). ADL, Activities of Daily Living; AUC, area under the curve; CFS, Clinical Frailty Scale; IADL, Instrumental Activities of Daily Living; MNA, Mini Nutritional Assessment; TUG, Timed Up and Go.
